# Correlation Between Maternal Smoking During Pregnancy and Dental Caries in Children: A Systematic Review and Meta-Analysis

**DOI:** 10.3389/froh.2021.673449

**Published:** 2021-06-16

**Authors:** Yongjin Zhong, Quan Tang, Bowen Tan, Ruijie Huang

**Affiliations:** State Key Laboratory of Oral Diseases, Department of Pediatric Dentistry, National Clinical Research Center for Oral Diseases, West China Hospital of Stomatology, Sichuan University, Chengdu, China

**Keywords:** pregnancy, tobacco, maternal smoking, dental caries, children

## Abstract

**Background:** Dental caries is a long-standing oral health problem for children all over the world. The available evidence shows that the association between maternal smoking during pregnancy and childhood caries is still controversial. Therefore, the aim of this systematic review and meta-analysis was to determine whether there was a correlation of prenatal smoking and dental caries in children.

**Methods:** PubMed, EMBASE, Cochrane, Web of Science, and Scopus databases were searched for observational studies assessing the relationship between maternal smoking during the pregnancy and childhood caries. According to the predesigned eligibility criteria and items, studies selection, and data extraction were conducted, respectively. The effect estimates were pooled using a fixed-effect model or a random-effect model. Newcastle-Ottawa Scale (NOS) was adopted to evaluate the methodological quality of the included studies. All analyses were carried out through Stata 12.0 software.

**Results:** Our systematic review included a total of 11 studies, of which 6 cross-sectional studies and 3 longitudinal studies were included in the final meta-analysis. The pooled estimates indicated maternal smoking during pregnancy was significantly associated with dental caries in children both in cross-sectional studies (OR = 1.57, 95% CI = 1.47–1.67) and longitudinal studies (RR = 1.26, 95% CI = 1.07–1.48). Sensitivity analyses confirmed the overall effect estimates were robust.

**Conclusions:** There is a significant correlation of maternal smoking during pregnancy and childhood caries. However, the causal relationship between them cannot be determined. More prospective and extensive studies on this theme is needed for verification. Even so, it is necessary for pregnant women and women of reproductive age to quit smoking. Strategies must be developed to raise public awareness about the impact of prenatal smoking on children's oral health.

## Introduction

According to The World Health Organization statistics, as of 2018, there are 1.337 billion people smoking, among which female smokers account for 244 million [1]. Tobacco use is responsible for lethal diseases such as laryngeal cancer, esophageal cancer, lung cancer and cardiovascular diseases [2]. And there is growing evidence that tobacco smoking causes numerous unfavorable effects on oral health. Factors in this regard include that tobacco may alter oral microenvironment [3], reduce saliva flow to impair the clearance effect, facilitate the occurrence of precancerous lesions [4], and act as a significant factor for oral cancer.

Regarded as a pandemic due to its worldwide distribution, dental caries causes severe consequences to human health. According to statistics, global untreated caries prevalence in primary and permanent dentition is 9 and 35%, respectively in 2010 [5]. Generally speaking, its mechanism is mainly in virtue of the bacteria residing in the dental plaque biofilm which decompose food to produce acid, resulting in demineralization of dental hard tissues. Children are vulnerable to dental caries due to their taste for sugar, and among all age group, the prevalence of dental caries peaks at age six [6]. Without timely treatment, dental caries can evolve into pulpitis and periapical inflammation, causing unbearable pain, restricted dietary intake and even tooth loss. Severe deciduous tooth caries can affect the development of tooth germs of the permanent teeth causing enamel hypoplasia while abnormal absorption of infected root can lead to disorderly eruption of permanent teeth [7].

Prenatal smoking is considered to be related to numerous undesirable perinatal health outcome in infants. As for the prevalence of tobacco use by sex in 2015, 40.3% males smoke compared with 9.5% in females [1]. Maternal smoking during pregnancy can be quite detrimental to fetal health, leading to fetal malformations, shortened gestational age, low birth weight, cleft lip and cleft palate [8]. Moreover, some researches have manifested that the occurrence of certain diseases in childhood may also be associated with prenatal smoking, which include childhood fractures [9], mental illness [10], and respiratory infections [11, 12].

The adverse effects of prenatal smoking on the health of offspring and the prevalence of childhood dental caries have led to hypothesis about a possible cause-and-effect relationship between them. Recently, although an increasing number of studies focused on the association between maternal smoking during pregnancy and childhood caries, their conclusions were not consistent [13–23]. Some studies suggested prenatal smoking could increase the risk of developing dental caries in children [13, 14, 16–19, 21, 22], while others did not observe any association between them [15, 20, 23]. These completely opposite results may be caused by various possible confounders because existing evidences suggest multiple factors like socioeconomic status, involuntary smoking, breastfeeding and its duration, maternal educational level and mode of delivery will affect the development of dental caries in children [19, 24–28]. Some studies have shown that children born in smoking households may be raised in a low socioeconomic level with insufficient nutrition [29] and have poorer oral hygiene with lower frequency of tooth brushing and more sugar intake [30, 31], which are conducive to the occurrence of dental caries.

Thus, to further explore whether there is a correlation of maternal smoking during pregnancy and dental caries in children, this systematic review with meta-analysis is performed.

## Methods

This systematic review was conducted based on the Preferred Reporting Items for Systematic Reviews and Meta-Analysis (PRISMA) [32].

### Eligibility Criteria

The following inclusion criteria were adopted: (a) cross sectional or longitudinal studies; (b) studies assessed the association between prenatal smoking and dental caries in children; (c) original data were presented in the studies or odds ratios (ORs), risk ratios (RRs), or hazard ratios (HRs) with corresponding 95% confidence intervals (CIs) calculated from study results were provided.

The exclusion criteria were as follows: (a) narrative reviews, case-reports, letters, comments, animal studies, *in vitro* studies and conference abstracts were excluded; (b) studies did not report the exposure of interest (maternal smoking during pregnancy) or the outcome of interest (childhood caries); (c) studies did not provide complete data; (d) studies were not published in English.

### Search Strategy and Studies Selection

In order to identify the relevant articles on the correlation of maternal smoking during the pregnancy and childhood caries, a comprehensive electronic search was carried out in the databases: PubMed, EMBASE, Cochrane, Scopus, as well as Web of Science databases up to March 29, 2021. The search term for PubMed was: ((((((((Pregnancy[MeSH Terms]) OR (gestation)) OR (pregnant)) OR (prenatal)) OR (antenatal)) OR (maternal)) AND ((((((((Tobacco smoking[MeSH Terms]) OR (smoking)) OR (cigar smoking)) OR (cigarette smoking)) OR (tobacco)) OR (tobacco products)) OR (cigar)) OR (cigarette))) AND (((((((Dental caries[MeSH Terms]) OR (dental decay)) OR (caries)) OR (teeth decay)) OR (carious lesions)) OR (carious dentin)) OR (dental white spot))) AND (((Child[MeSH Terms]) OR (children)) OR (childhood)). Search terms for other databases were showed in Supplementary Table 1.

In the studies selection process, duplicate articles were removed first. Then, two authors (YZ and QT) independently read the titles and abstracts to screen out the articles that need to be fully assessed. Finally, the studies which met the eligibility criteria were included after full-text evaluation. Disagreements were resolved through discussion or consultation with a third author (BT).

### Data Extraction

The following items were extracted from each of the studies included: first authors, year of publication, data source, country, study design, sample size, age of children, exposure assessment, as well as outcome assessment. When available, we collected the risk estimates (ORs, RRs, or HRs) with their 95% CIs for the correlation of prenatal smoking and childhood caries from the included articles. If not possible, we used the raw data reported in the studies to calculate the corresponding effect size and the 95% CIs. The above data were repeatedly checked by the two authors (YZ and QT).

### Quality Evaluation

The Newcastle-Ottawa Scale (NOS) was adopted to assess the methodological quality of the included studies [33]. The scoring criteria of the scale were based on three aspects: selection (four scoring items), comparability (two scoring items), and exposure/outcome (three scoring items). Each item corresponded to one point. 0–3, 4–6, and 7–9 points, respectively, represented low, medium and high quality of studies.

### Statistical Analysis

In the present meta-analysis, OR and RR associated 95% CI were selected to report the results of the included cross-sectional studies and longitudinal studies, respectively. I^2^ statistics and Q test were used to assess the heterogeneity of the included studies [34]. Among them, *P* > 0.1 (Q test) or I^2^ <50% was identified as low heterogeneity. The choice of pooling model was based on the level of heterogeneity. When the heterogeneity was significant, a random-effect model was adopted to summarized the results, otherwise, the fixed-effect model was selected. To investigate the factors which could influence the pooled results, we performed subgroup analyses based on virous study characteristics (region, children's age, sample size, and the method of caries assessment). Sensitivity analyses were conducted to confirm the robustness of the results using a one-study omitted approach. Funnel plot, Begg's test and Egger's test were used to detect the publication bias among the included studies [35, 36]. All analyses were carried out by Stata version 12.0 (Stata Corporation, College Station, TX, USA).

## Results

### Selection of the Studies

**Figure 1:**
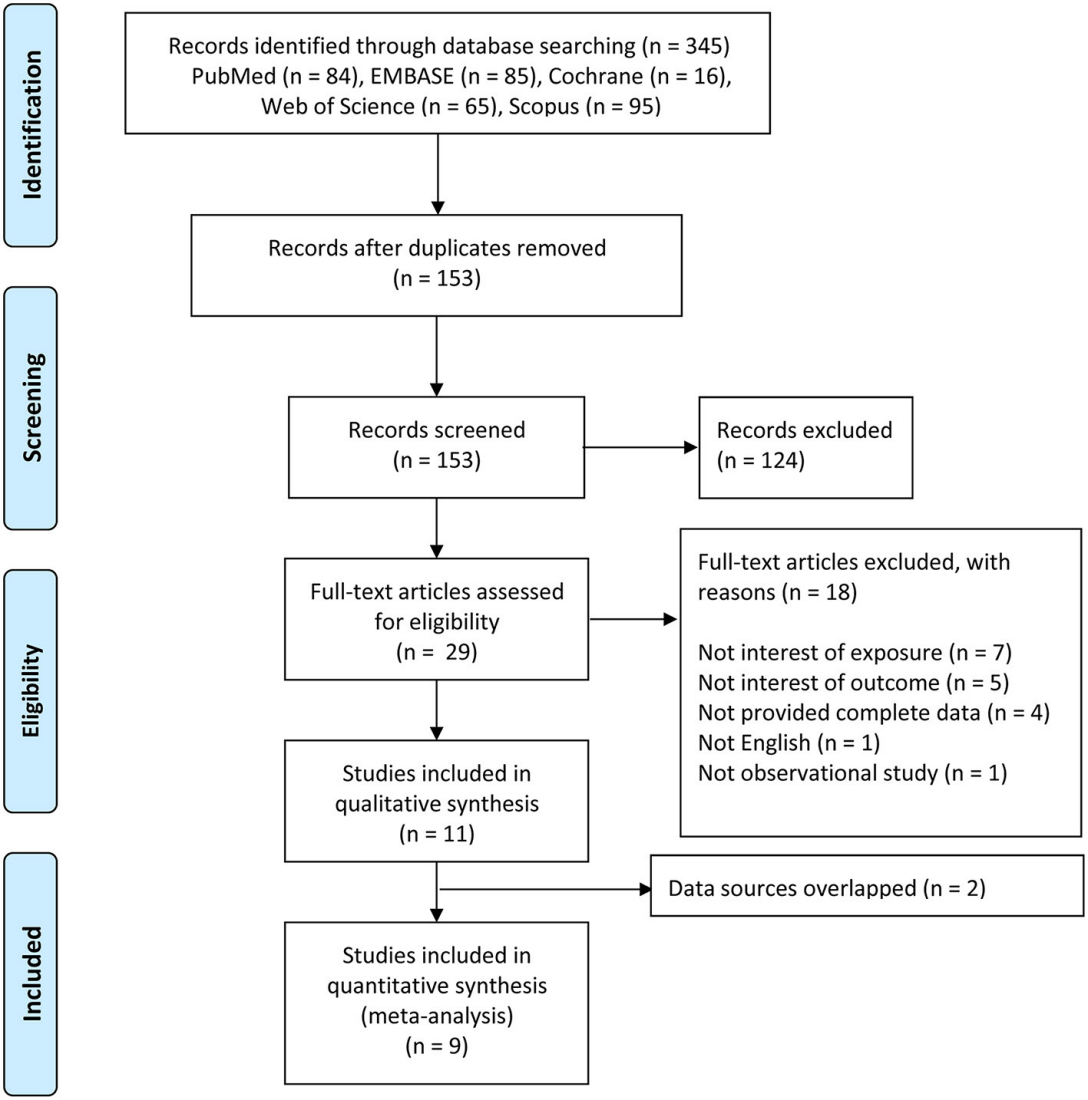
Flow chart of search process and reasons for exclusion.

A total of 345 records were retrieved though database searching: PubMed (*n* = 84), EMBASE (*n* = 85), Cochrane (*n* = 16), Scopus (*n* = 95), and Web of Science (*n* = 65). At the beginning, 192 duplicates were excluded. After screening the titles and abstracts, 124 records were removed. The remaining 29 full-text articles were further evaluated according to eligibility criteria. Ultimately, 11 studies were included in our qualitative synthesis (Figure 1) [13–23]. Of these, 9 studies were included in the meta-analysis [14–17, 19–23]. Two articles [Akinkugbe [13] and Julihn et al. [18]] were excluded because their data sources overlapped with the other articles, and we selected the articles [Akinkugbe et al. [14] and Julihn et al. [19]] with larger sample size or more complete data.

### Characteristics of the Included Studies

The detailed characteristics of the included studies were summarized in Table 1. Among the included studies, six were cross-sectional studies [15, 17, 19–22] and five were longitudinal studies [13, 14, 16, 18, 23]. For the region, six studies came from Europe [13–15, 18–20]; three studies were conducted in Asia [21–23]; one was from the United States [17]; and one was carried out in Australia [16]. In terms of exposure assessment, most studies using the self-reported information by pregnant women. For the outcome assessment, the data of dental examinations were used in the majority of studies. However, the specific caries detection methods varied from study to study: three studies only used visual examination [21–23]; three studies adopted tactile/visual examination [15, 17, 20]; and one study reported data on childhood caries also came from the results of radiographic examination [19]. Moreover, the evaluation results of the quality of each study were presented in Table 2. Most studies were of high quality, and there were no low-quality studies.

**Table 1:** Characteristics of the included studies.

**Author (year)**	**Data source**	**Study design/country**	**Sample**	**Children's age**	**Exposure assessment**	**Outcome assessment**	**Effect estimates (95% CI)**	**Association**
Akinkugbe [13]	The 1991/92 Avon Longitudinal Study of Parents and Children (ALSPAC)	L England	1,429 children	5y	Self-report	Clinical oral examinations	RR: 1.60 (1.09–2.32)	Yes
Akinkugbe et al. [14]	The 1991/92 Avon Longitudinal Study of Parents and Children (ALSPAC)	L England	1,429 children	5y	Self-report	Clinical oral examinations	RR: 1.30 (1.08–1.58)	Yes
Borowska-Struginska et al. [15]	Randomly selected schools and kindergartens in the city of Łódz	CS Poland	1,131 children	5–13y	Self-report	Dental examinations (visual/tactile examination)	OR^*^: 1.42 (0.95–2.12)	No
Claudia et al. [16]	The Longitudinal Study of Indigenous Children (LSIC)	L Australia	1,687 children	0.5–6y	Self-report	Parent-reported dental caries	RR: 1.42 (1.20–1.68)	Yes
Iida et al. [17]	The National Health and Nutrition Examination Survey (NHANES)	CS United States	1,576 children	2–5y	Self-report	Dental examination (visual/tactile examination)	OR: 1.68 (1.01–2.79)	Yes
Julihn et al. [18]	The Public Health Care Administration in Stockholm and the national registers at the National Board of Health and Welfare and Statistics	L Sweden	15,538 children	13–19y	Self-report	Database	OR: 1.33 (1.22–1.44)	Yes
Julihn et al. [19]	The Public Health Care Administration in Stockholm and the national registers at the National Board of Health and Welfare and Statistics	CS Sweden	65,259 children.	3–7y	Database	Clinical and radiographic examinations	OR: 1.56 (1.42–1.63)	Yes
Majorana et al. [20]	The two obstetric wards of Brescia Hospital	CS Italy	2,395 children.	2–2.5y	Self-report	Dental examinations (visual/tactile examination)	OR^*^: 1.42 (0.92–2.21)	No
Tanaka et al. [21]	The Kyushu Okinawa Child Health Study (KOCHS)	CS Japan	6,412 children	3y	Self-report	Dental examination (visual examination)	OR: 1.70 (1.15–2.48)	Yes
Tanaka et al. [22]	The Fukuoka Child Health Study (FCHS)	CS Japan	2,015 children	3y	Self-report	Dental examination (visual examination)	OR^*^: 2.03 (1.33–3.09)	Yes
Tanaka et al. [23]	The Kobe Offspring Study	L Japan	76,920 children	3y	Self-report	Dental examination (visual examination)	HR: 1.10 (0.97–1.25)	No

* *means these effect estimates were calculated using raw data*.

*L, longitudinal study; CS, cross-sectional study; OR, odds ratio; RR, risk ratio; HR, hazard ratio*.

**Table 2:** The methodological quality of the included studies.

**Study**	**Selection**	**Comparability**	**Exposure/Outcome**	**Total scores**
Akinkugbe [13]	^****^	^**^	^***^	9
Akinkugbe et al. [14]	^****^	^**^	^***^	9
Borowska-Struginska et al. [15]	^****^		^**^	6
Claudia et al. [16]	^***^	^**^	^***^	8
Iida et al. [17]	^****^	^**^	^**^	8
Julihn et al. [18]	^****^	^**^	^***^	9
Julihn et al. [19]	^****^	^**^	^***^	9
Majorana et al. [20]	^****^		^**^	6
Tanaka et al. [21]	^****^	^**^	^**^	8
Tanaka et al. [22]	^****^	^**^	^**^	8
Tanaka et al. [23]	^****^	^**^	^**^	8

** *Two points*;

*** *three points; and*

**** *four points*.

### Meta-Analysis

**Figure 2:**
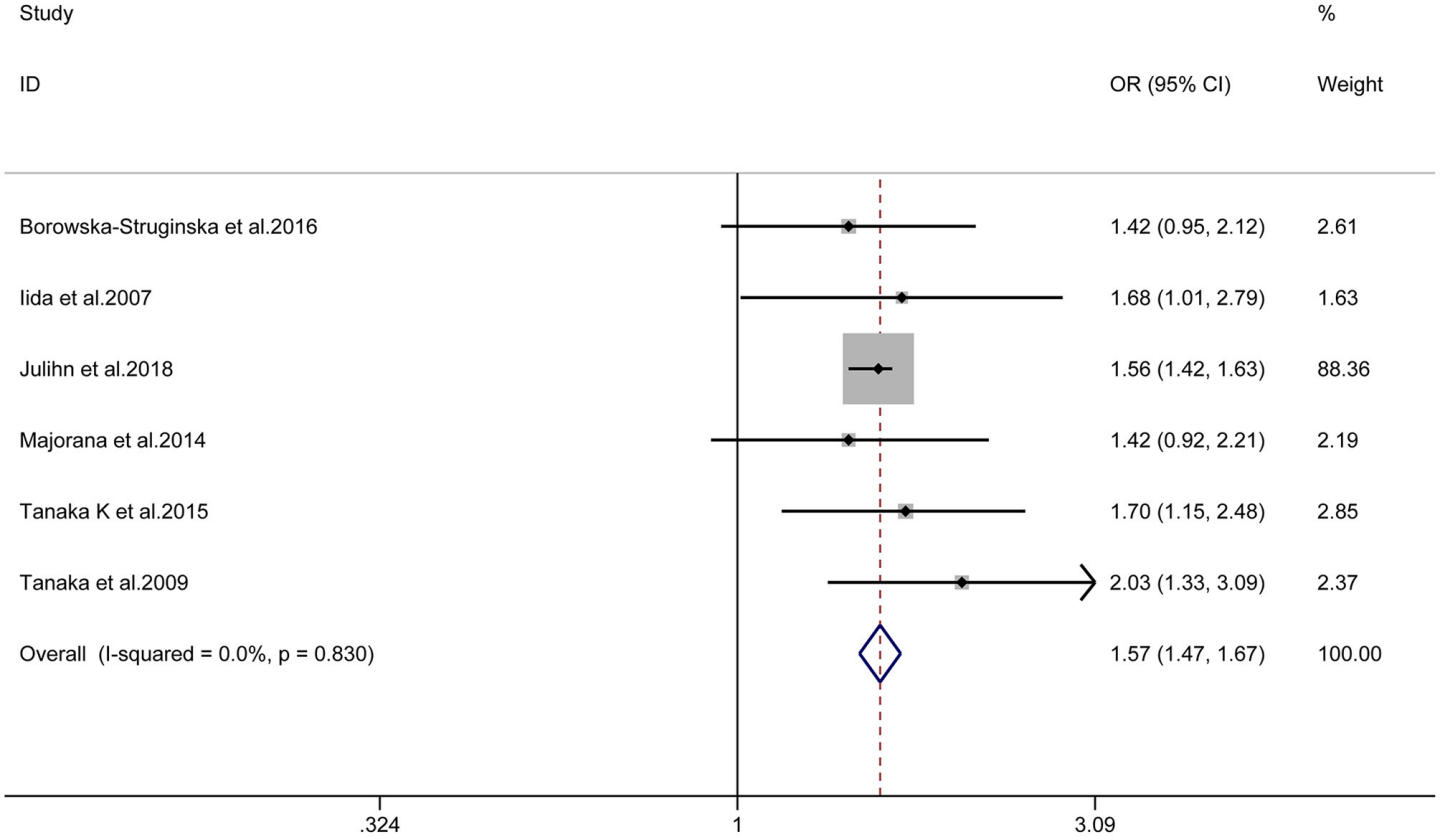
Forest plot of maternal smoking during pregnancy and childhood caries (cross-sectional studies).

**Figure 3:**
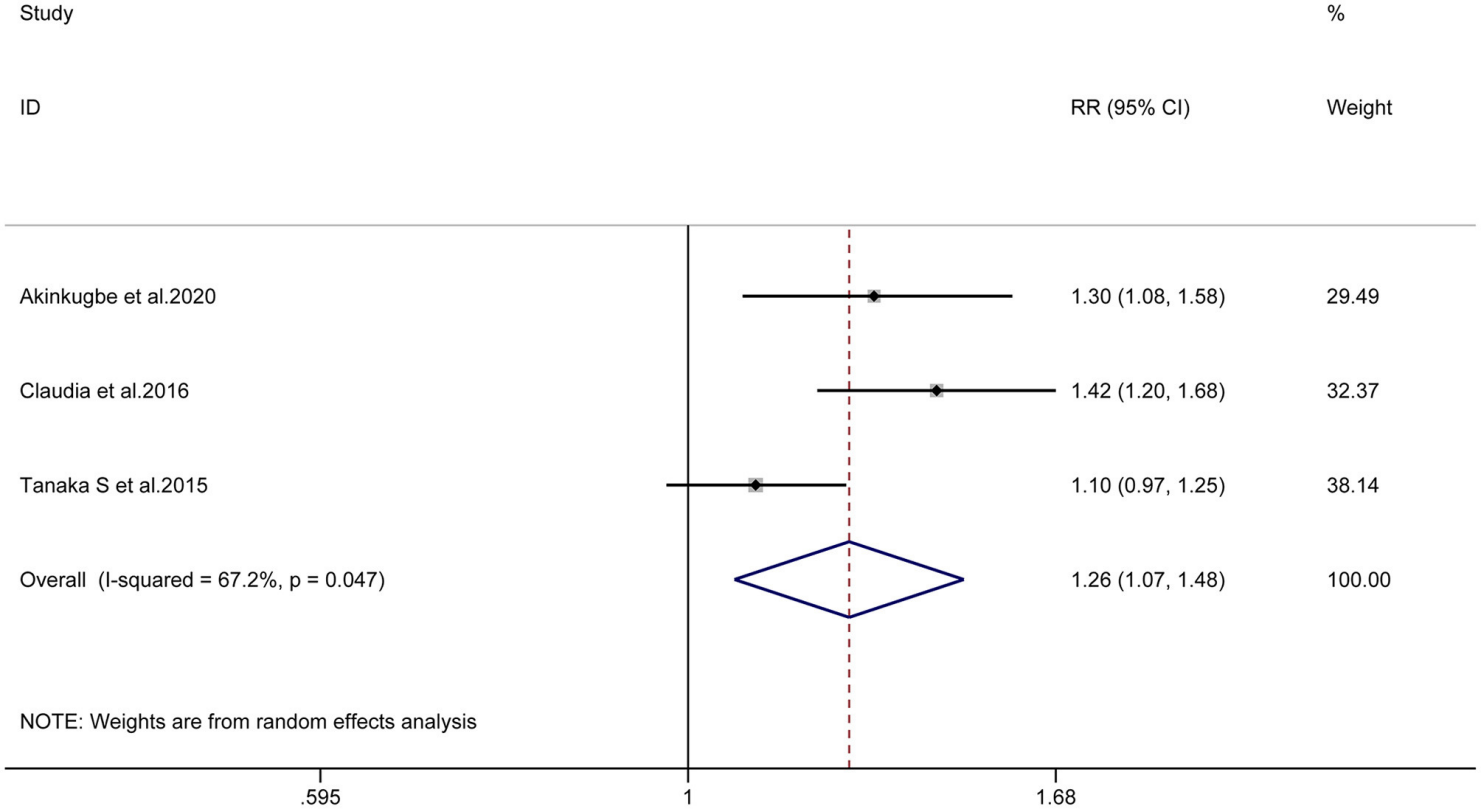
Funnel plot of the included cross-sectional studies on the association between prenatal smoking and dental caries in children.

Six cross-sectional studies [15, 17, 19–22] and three longitudinal studies [14, 16, 23] were included in the final quantitative synthesis, and we analyzed their results separately. The pooled result of the cross-sectional studies indicated maternal smoking during pregnancy was significantly associated with the risk of dental caries in children (OR = 1.57, 95% CI = 1.47–1.67, I^2^ = 0; Figure 2). The sensitivity analyses suggested that our meta-analysis results were robust (Table 3). The funnel plot (Figure 3), Begg's test (*P* = 1.00) and Egger's test (*P* = 0.55) were indicative of no publication bias for the correlation of prenatal smoking and childhood caries.

**Table 3:** The results of subgroup and sensitivity analyses.

**Subgroup**	**No. of studies**	**OR (95% CI)**		**Heterogeneity**	
		**OR**	**95% CI**	***I^**2**^***	***P***
**Overall**	6	1.57	1.47–1.67	0	0.830
**Study region**					
Europe	3	1.55	1.45–1.66	0	0.832
Asia	2	1.84	1.39–2.45	0	0.542
United States	1	1.68	1.01–2.79	NA	NA
**Children's age**					
≤ 3	3	1.71	1.34–2.16	0	0.515
>3	3	1.56	1.46–1.67	0	0.865
**Sample size**					
≤ 3,000	4	1.61	1.30–2.01	0	0.598
>3,000	2	1.56	1.46–1.67	0	0.666
**Dental examinations**					
Visual examination	2	1.84	1.39–2.45	0	0.542
Visual/tactile examination	3	1.48	1.15–1.91	0	0.855
Clinical and radiographic examination	1	1.56	1.46–1.67	NA	NA
**Sensitivity analyses**					
Excluded Borowska-Struginska et al. [15]	5	1.57	1.47–1.68	0	0.755
Excluded Iida et al. [17]	5	1.57	1.47–1.67	0	0.724
Excluded Julihn et al. [18]	5	1.63	1.35–1.98	0	0.748
Excluded Majorana et al. [20]	5	1.57	1.47–1.68	0	0.748
Excluded Tanaka et al. [21]	5	1.57	1.47–1.67	0	0.743
Excluded Tanaka et al. [22]	5	1.56	1.46–1.66	0	0.956

*NA, not available*.

**Figure 4:**
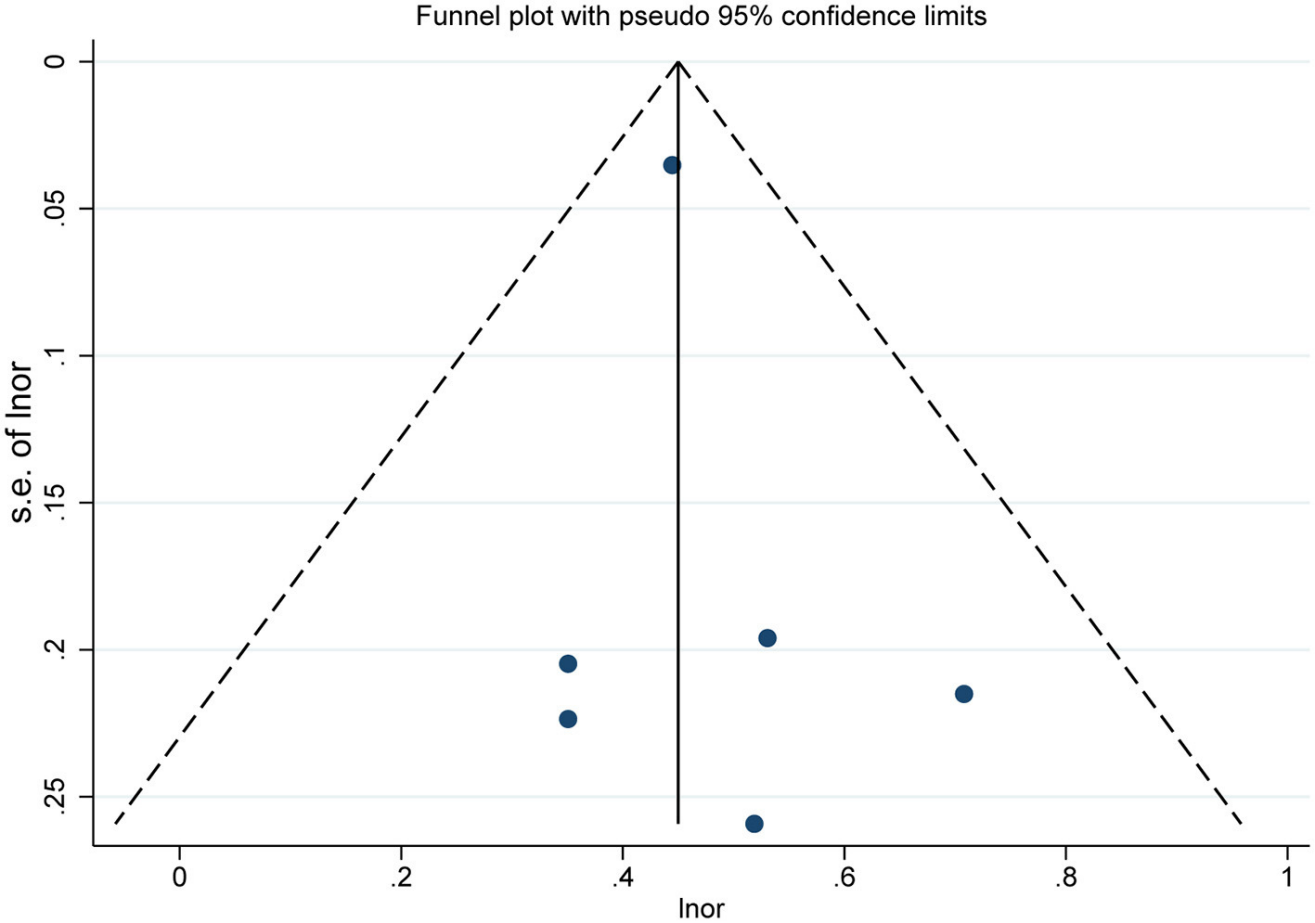
Forest plot of maternal smoking during pregnancy and childhood caries (longitudinal studies).

Similarly, the overall meta-analysis of longitudinal studies also showed a correlation between prenatal smoking and childhood caries (RR = 1.26, 95% CI = 1.07–1.48, I^2^ = 67.2%; Figure 4). Due to the limitation of the number of articles, we did not subsequently perform subgroup analysis, sensitivity analysis and publication bias evaluation of these longitudinal studies.

### Subgroup Analysis

Subgroup analysis was conducted according to different characteristics of the included studies. Table 3 presented the pooled results of each subgroup. In terms of the study region, the results showed that there was a stronger association between tobacco smoking during pregnancy and the risk of childhood caries in Asia (OR = 1.84, 95% CI = 1.39–2.45). Compared to children older than 3 (OR = 1.56, 95% CI = 1.46–1.67), children younger than 3 (OR = 1.71, 95% CI = 1.34–2.16) were more likely to develop dental caries after maternal smoking during pregnancy. When subgroup analysis stratified by the methods of caries examination, the pooled risk estimates were different from each other (visual examination: OR = 1.84, 95% CI = 1.39–2.45; tactile/visual examination: OR = 1.48, 95% CI = 1.15–1.91; clinical and radiographic examination: OR = 1.56, 95% CI = 1.46–1.67). Furthermore, there was little difference between the results of sample size ≤ 3,000 and sample size > 3,000.

## Discussion

The objective of this study was to evaluate whether prenatal smoking was associated with childhood caries. Our meta-analysis results indicated that the existence of a correlation between maternal smoking during pregnancy and the risk of developing dental caries in children. Sensitivity analyses confirmed the results were stable, and no publication bias was observed among the included studies.

The biological cues clarifying the association between prenatal smoking and dental caries in children have not been identified. Some previous studies may provide insights to understand the potential relationship but more experimental researches are required to better illustrate the definite mechanism. One possible cue is that tobacco smoke can be pernicious to the formation or mineralization of the deciduous teeth. The formation and mineralization of dentin is accomplished by odontoblast cells, which are then located in the pulp as part of the pulp cells capable of inducing reparative dentinogenesis. Yanagita et al. demonstrated that nicotine could inhibit mineralization of human dental pulp cells, thus the dentin matrix synthesis and mineralization were reduced in smokers [37], and teeth with hypomineralization or impaired ability to generate restorative dentin are susceptible to dental caries. Moreover, it has also been reported that nicotine can facilitate the biofilm formation and metabolic activity of *Streptococcus mutans* which acts as a key pathogen contributing to dental caries [38]. The adherence of *S. mutans* is also reported to be enhanced in the presence of nicotine [39]. However, these studies cannot confirm whether maternal smoking transmits the above adverse effects to the fetus. A more convincing study demonstrated through animal experiments that offspring of rats exposed to passive smoking during pregnancy were more likely to suffer from dental caries. In this study, delayed development of dental hard tissues in offspring was observed, as evidenced by inhibited morphologic development and suppressed mineralization [40]. Another potential mechanism that mediates prenatal smoking causing dental caries in children is vitamin D deficiency, as vitamin D is related to the tooth mineralization. Previous study has shown that lower maternal intake of vitamin D could increase the risk of dental caries in children [41]. Besides, it has been observed that the serum concentration of vitamin D is inversely proportional to serum cotinine levels [42], which may indicate that prenatal smoking suppresses the mineralization of deciduous tooth by affecting maternal vitamin D content.

In addition to these etiological evidences, there are grounds to assume that the association between prenatal smoking and caries in children is mainly due to socioeconomic factors. Generally, children with lower living standards exhibit higher caries rates [29–31]. It should be noted that post-partum smoking or household smoking has also been shown to be a possible predictor of dental caries in offspring since children would involuntarily inhale tobacco smoke. Tanaka et al. [23] observed 12,729 caries cases, and they found that in families with no smokers, the risk of dental caries for children at the age of three was 14.0%, while the risk of caries for children exposed to tobacco smoke was 27.6%. Goto et al. [43] reported that compared with non-exposure to tobacco smoke, exposure to maternal smoking for more than 3 pack-years and exposure to smoking by all family members for more than 5 pack-years were significantly associated with the risk of dental caries in preschool children. González-Valero et al. [24] conducted a meta-analysis of related articles, and the results suggested that the incidence risk of dental caries in children exposed to tobacco smoking during infancy was 1.7 times higher than that of children who were not exposed to tobacco smoking. The development of childhood caries is also potentially related to other characteristic of mothers. Some studies have speculated that breastfeeding may affect the risk of dental caries in offspring. Hagg et al. [25] found that children breastfed > 24 months had a higher prevalence of dental caries and mean dmfs (decayed/missing/filled surface), compared with children without breastfeeding. However, Bernabé et al. [26] reported that there was no correlation between the duration of breastfeeding and dental caries in children. Boustedt et al. [28] found that children born by cesarean section had a 2-fold increase in the risk of dental caries at the age of 5 compared to the children delivered vaginally, which means that the mode of delivery has a significant impact on childhood caries. In addition, factors such as maternal education level [27], maternal obesity [19], and maternal age at delivery [44] which may be associated with the risk of dental caries in children have been reported. Therefore, it is very important to strictly control the maternal characteristics in future studies on the association between maternal smoking during pregnancy and dental caries in children to avoid these factors from interfering with the results.

A previous study conducted a systematic review on the association between cigarette smoking during pregnancy and childhood caries [45], however, the study did not reach a definite conclusion. Compared with it, our study formulated stricter inclusion criteria and updated more articles, and we confirmed that prenatal tobacco smoking was statistically significant associated with offspring caries through meta-analysis. Furthermore, we performed subgroup analyses based on the different characteristics of the included studies. The subsets of children younger than 3, visual examination and studies from Asia had higher effect estimates, which meant there were stronger association between maternal smoking during pregnancy and childhood caries among these subgroups. Given the small number of articles in each subgroup, this conclusion may not be reliable. More research is needed to confirm this finding. Nevertheless, we still need to pay more attention to the dental health of young children, use unified standards and methods for childhood caries examination, and use precise radiological examination when necessary.

There is a dose-response relationship between tobacco smoking during pregnancy and dental caries in children. A study reported that children whose mothers smoked ≥ half pack/day during pregnancy were more likely to develop dental caries compared to the children whose mothers smoked < half pack/day [14]. For every 10 cigarettes smoked during pregnancy, the risk of childhood caries increased by 20% [14]. It is worth noting that the duration of smoking habits may affect the association between prenatal smoking and childhood caries. Tanaka et al. [22] found a correlation of maternal smoking and childhood caries among children whose mothers smoked throughout pregnancy while the association was not statistical significance among children whose mothers stopped smoking at some time during pregnancy. Moreover, considering that pregnancy can be roughly divided into three stages, some studies have explored the impact of maternal smoking at different pregnant stages on dental caries in offspring. Akinkugbe [13] reported tobacco smoking during the third trimester leaded a higher risk of childhood caries. However, Tanaka et al. [21] observed there was a closer association between maternal smoking during the first trimester than other stages.

Some limitations cannot be ignored in the present systematic review and meta-analysis. (a) Most of the study designs of the articles were cross-sectional studies in our meta-analysis and therefore the causal relationship between maternal smoking during pregnancy and childhood caries could not be determined. Though we subsequently pooled the results of the included three longitudinal studies [14, 16, 23], the hypothesis that tobacco smoking during pregnancy could be a possible risk factor of childhood dental caries was not convincing due to the lack of longitudinal articles. So more prospective longitudinal studies are needed to prove this conjecture; (b) Many potential confounding factors could affect the results. Though the majority of the included studies adjusted some main confounders (e.g., gender, toothbrushing frequency, and household income) and provided the adjusted effect estimates, few studies adjusted for maternal obesity, maternal age at delivery, and passive smoking. Besides, some articles did not report the adjusted effect estimates [15, 20, 22]. Thus, we could only calculate rough effect values based on the raw data, which may affect the final the overall results. Future research on this topic should be better designed to minimize the influence of possible confounding factors on the results. (c) Majorana et al. [20] investigated the prevalence of childhood caries with different severity levels (low, moderate, and high) after maternal smoking during pregnancy. However, we did not analyze the associations among caries severity levels owing to the lack of relevant data. (d) Self-reported information was adopted to assess the exposure in most of the included studies. Of which the biggest drawback was that it could cause response bias and affect the final meta-analysis results.

## Conclusion

To sum up, the present systematic review and meta-analysis indicated there is an association between maternal smoking during pregnancy and dental caries in children. However, this correlation is also affected by various confounding factors such as post-natal and household smoking exposure, breastfeeding and its duration, maternal age at delivery and mode of delivery. Therefore, more prospective studies with large-scale population and appropriate methodology for adjusting covariates are required in the future to further explore the causal relationship between prenatal smoking and childhood dental caries. Nevertheless, it is very necessary for pregnant women to quit smoking. Oral healthcare providers should carry out relevant publicity work on a regular basis to raise the awareness pregnant women, women of reproductive age and their families about the effects of maternal smoking during pregnancy on children's oral health.

## Data Availability Statement

The original contributions presented in the study are included in the article/Supplementary Material, further inquiries can be directed to the corresponding author/s.

## Author Contributions

YZ and RH contributed to conception and design of the study and wrote the first draft of the manuscript. YZ and QT organized the database. YZ performed the statistical analysis. YZ, QT, and BT wrote sections of the manuscript. All authors contributed to manuscript revision, read, and approved the submitted version.

## Conflict of Interest

The authors declare that the research was conducted in the absence of any commercial or financial relationships that could be construed as a potential conflict of interest.
